# Supplementary material for: Prediction of symptomatic and asymptomatic bacteriuria in spinal cord injury patients using machine learning
Source: Microbiome. 2025 Nov 27;13:246. doi: 10.1186/s40168-025-02213-8 (PMC12661733; doi:10.1186/s40168-025-02213-8)
Supplement: Supplementary file 2 — Supplementary Material 1. [file 40168_2025_2213_MOESM1_ESM.pdf]

Supplementary Information

Prediction of symptomatic and asymptomatic bacteriuria in spinal cord injury patients using machine learning

M. Mozammel Hoque[1], Parisa Noorian[1,] Gustavo Espinoza-Vergara[1], Joyce To[1], Dominic Leo[1], Priyadarshini Chari[2], Gerard Weber[3], Julie Pryor[3,4], Iain G. Duggin[1], Bonsan B. Lee[5,6], Scott A. Rice[1,7*], Diane McDougald[1*]

[1]Australian Institute for Microbiology & Infection, University of Technology Sydney, Sydney, NSW, Australia.

[2]Spinal cord injury unit, Royal North Shore Hospital, NSW, Australia.

[3]Royal Rehab Group, Sydney, NSW, Australia.

[4]Susan Wakil School of Nursing and Midwifery, University of Sydney, Sydney, Australia

[5]Department of Spinal and Rehabilitation Medicine, Prince of Wales Hospital, Sydney, NSW, Australia.

[6]Neuroscience Research Australia (NEURA), Sydney, NSW, Australia.

[7]CSIRO, Microbiomes for One Systems Health, Agriculture & Food, Westmead NSW.

*Correspondence to: diane.mcdougald@uts.edu.au and Scott.Rice@csiro.au

Running Title: Machine learning based prediction of bacteriuria.

Keywords: Spinal cord injury, Bacteriuria, Urinary tract infections, 16S rRNA, Catheter, Urine, Machine learning, Prediction.

Content:

Supplementary Fig. S1-10

Supplementary Table S1-8

Supplementary Figures

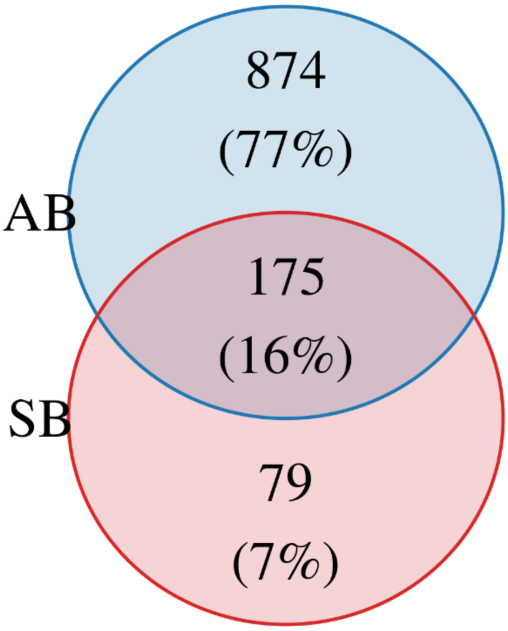

Supplementary Fig. S1. Venn diagram showing the unique and shared ASVs between asymptomatic (AB) and symptomatic (SB) bacteriuria groups.

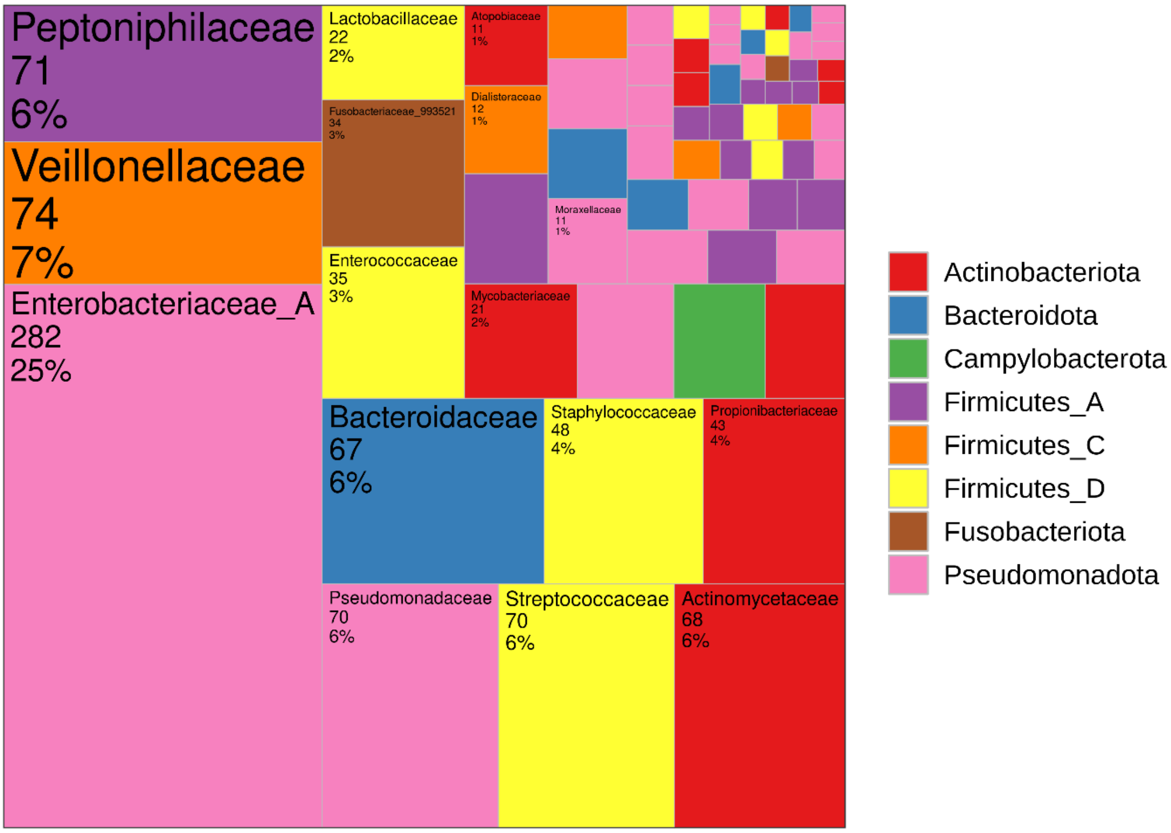

Supplementary Fig. S2. Treemap showing the fraction of ASVs assigned to Family. The number and percentages of ASVs belong to each family are denoted inside of each box. The colors depict corresponding phyla.

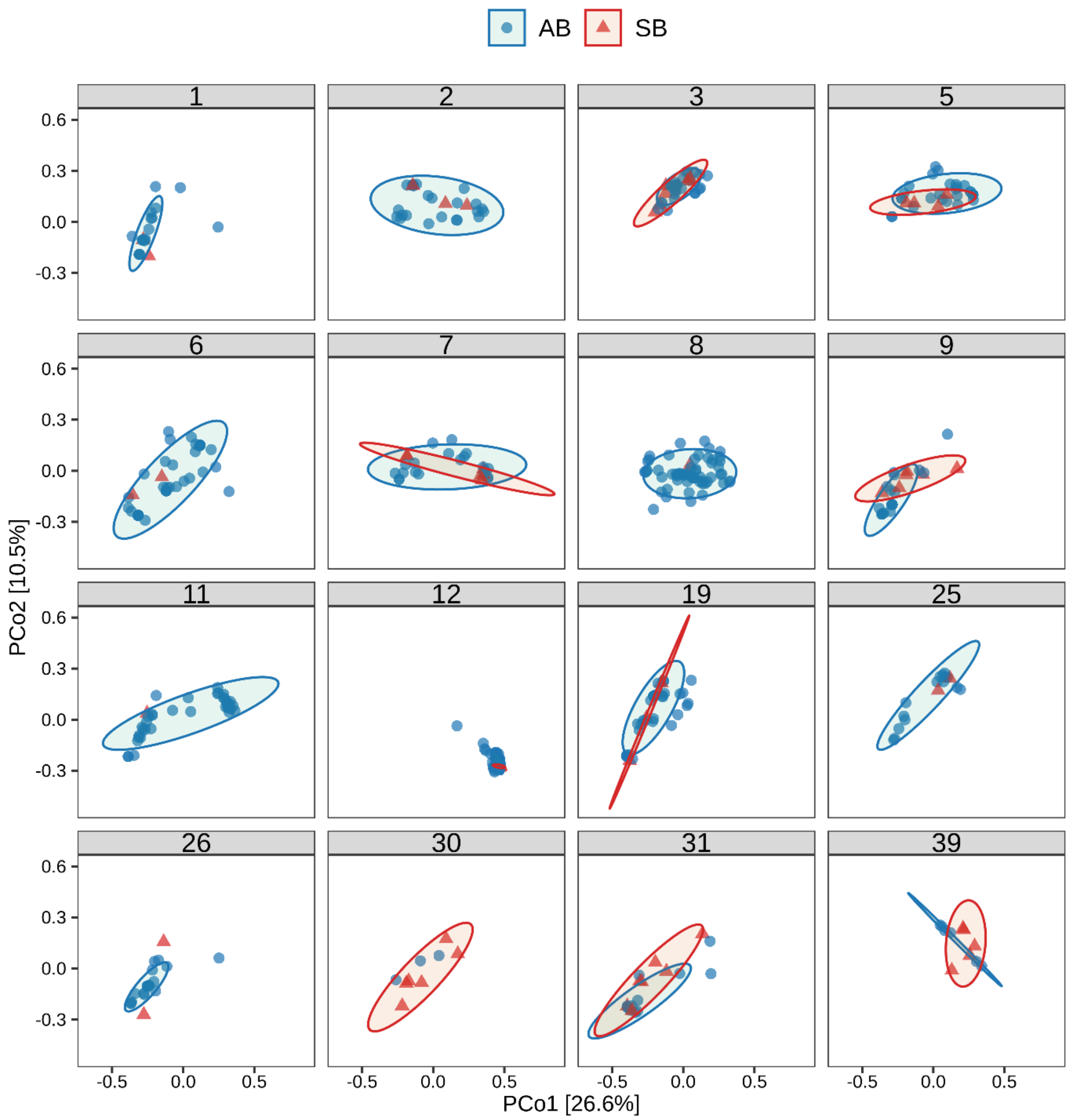

Supplementary Fig. S3. Beta diversity (unweighted unifrac) analysis in participants with at least one symptomatic bacteriuria event.

Principal coordinates analyses (PCoA) of beta-diversity between asymptomatic (AB, blue) and symptomatic (SB, red) bacteriuria group based on unweighted unifrac distance matrices. Each dot represents an individual sample. Participants with at least one SB event were included in this analysis. Ellipses are only drawn if there are 3 or more data points of the same group. Ellipses boundaries covers 95% of the data points.

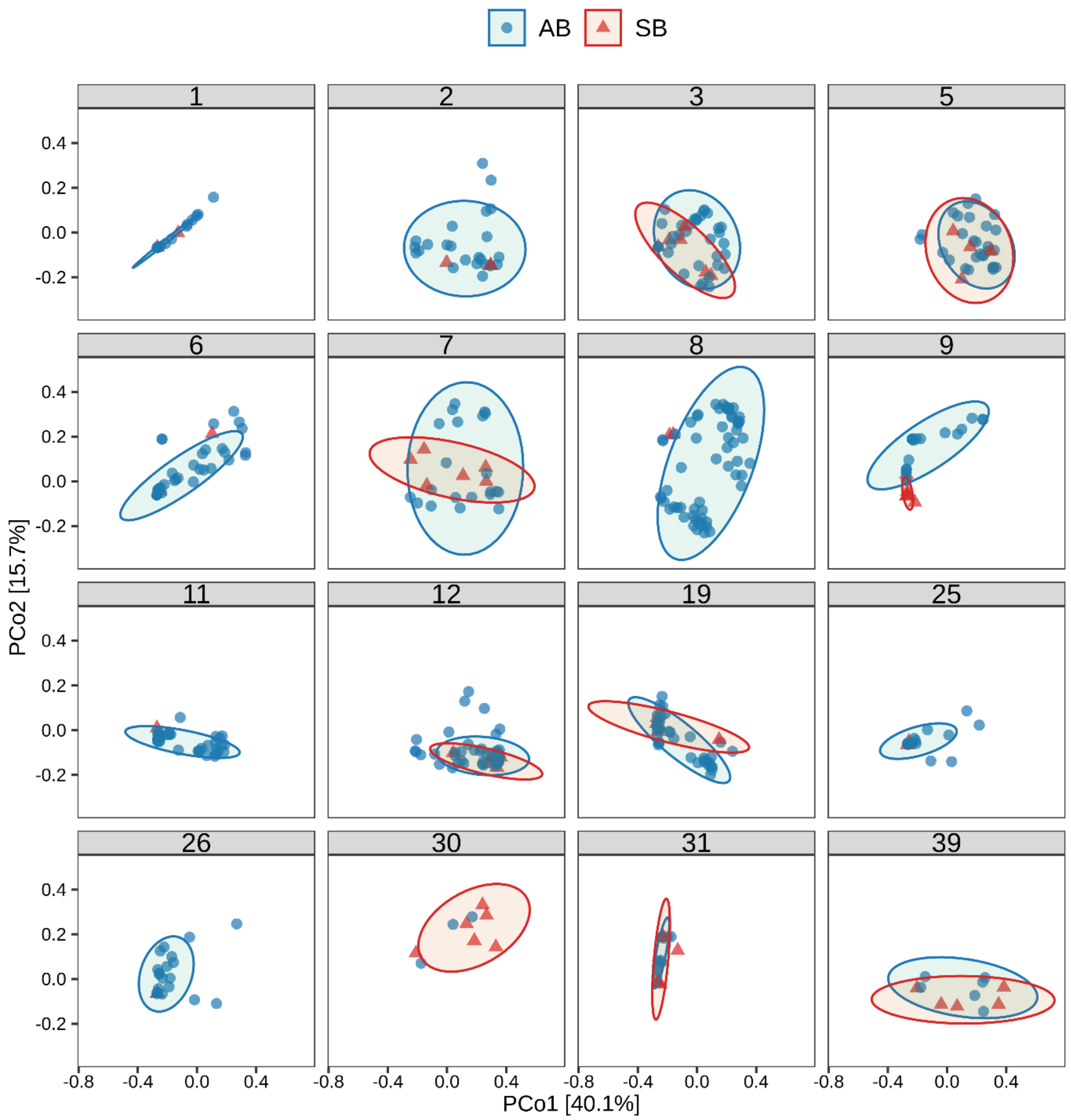

Supplementary Fig. S4. Beta diversity analysis (weighted unifrac) in participants with at least one symptomatic bacteriuria event.

Principal coordinates analyses (PCoA) of beta-diversity between asymptomatic (AB, blue) and symptomatic (SB, red) bacteriuria group based on weighted unifrac distance matrices. Each dot represents an individual sample. Participants with at least one SB event were included in this analysis. Ellipses are only drawn if there are 3 or more data points of the same group. Ellipses boundaries covers 95% of the data points.

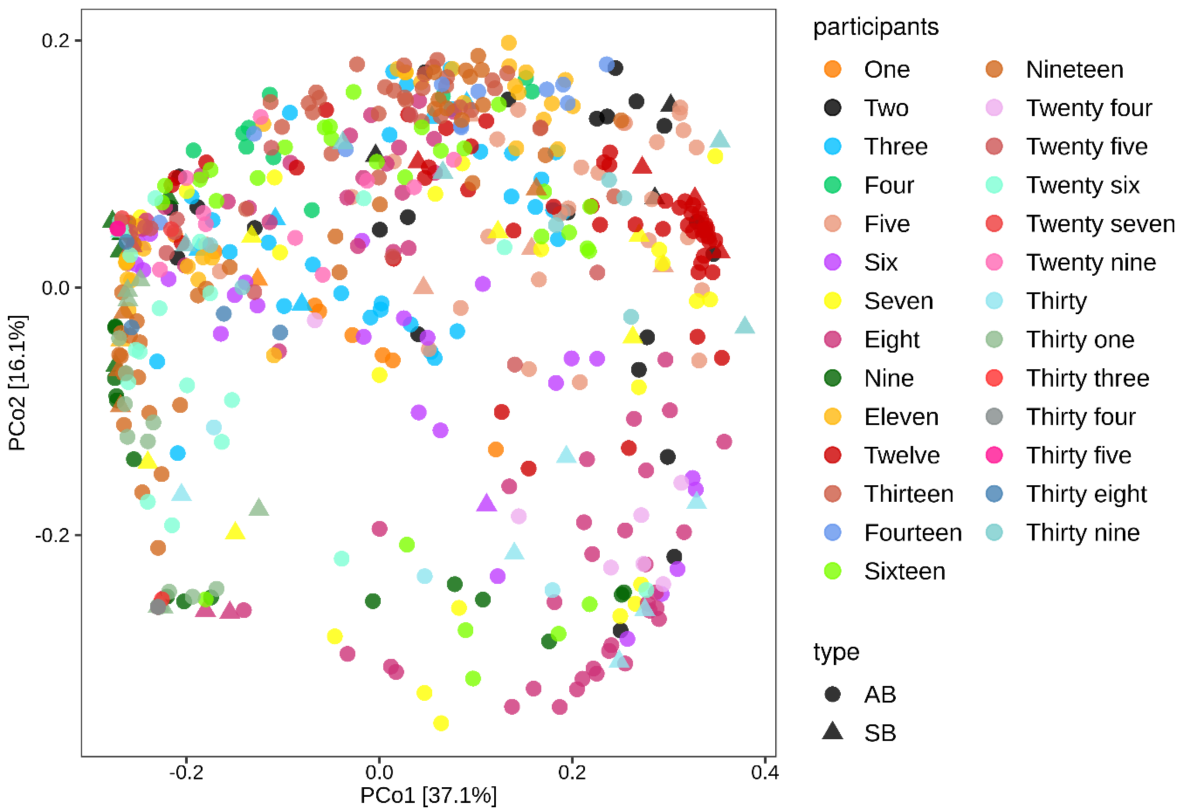

Supplementary Fig. S5. Principal coordinates analyses (PCoA) of beta-diversity depicting participant level variation based on weighted unifrac distance matrices. Each dot represents an individual sample. PCoA figure visualizes AB and SB by shape and colored by participants.

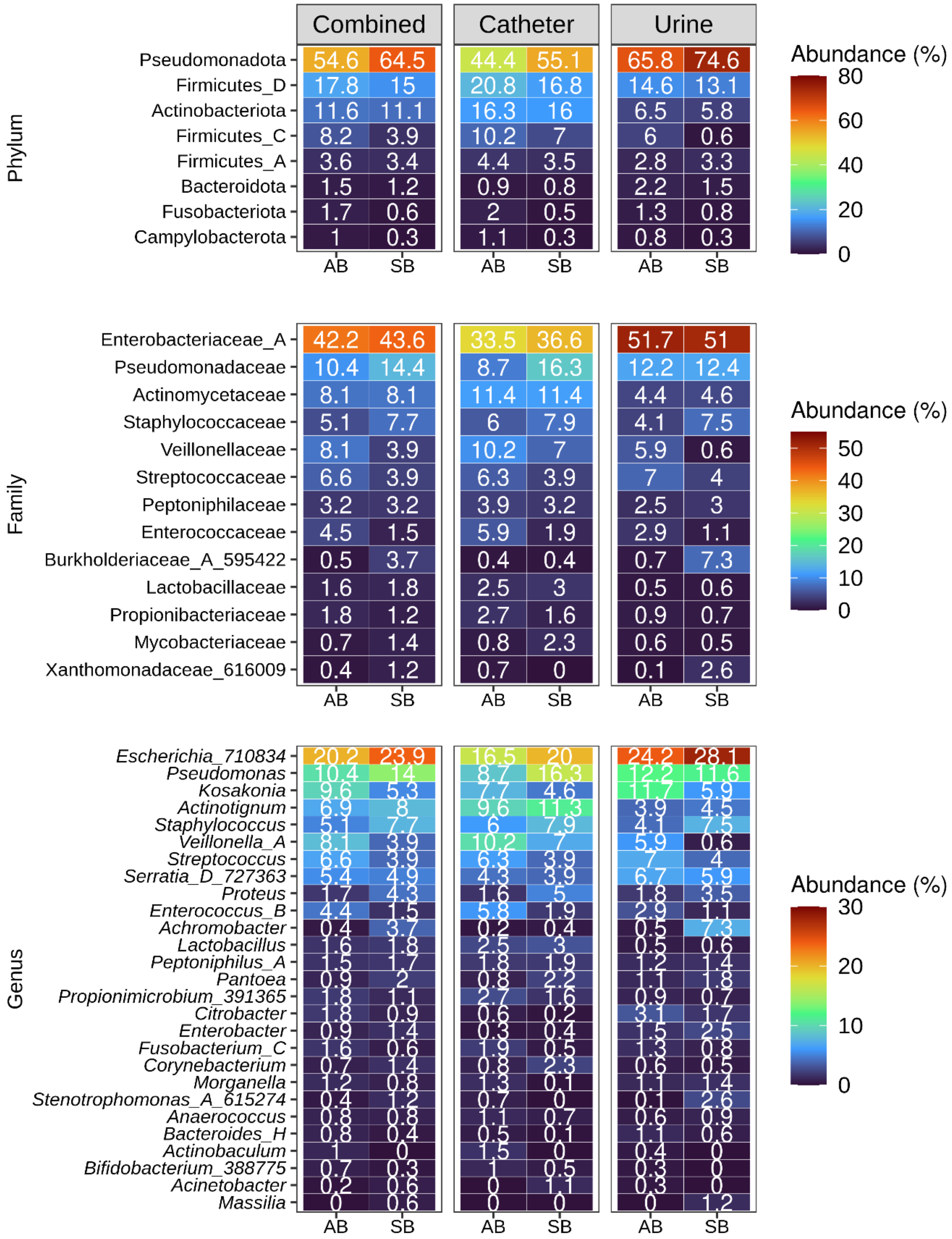

Supplementary Fig. S6. Overview of taxonomic composition in asymptomatic and symptomatic bacteriuria groups across combined, catheter and urine dataset.
The colour heatmaps depicting mean relative abundances in percentages ranging from blue (low abundance) to red (high abundance) grouped by phylum, family, and genus. The numbers inside heatmaps show mean relative abundance of corresponding taxa indicated in y-axis across asymptomatic bacteriuria (AB) and symptomatic (SB) groups. Genus are shown if their mean relative abundances in any of the group was more than 1. Taxa (rows) are sorted based on the abundances.

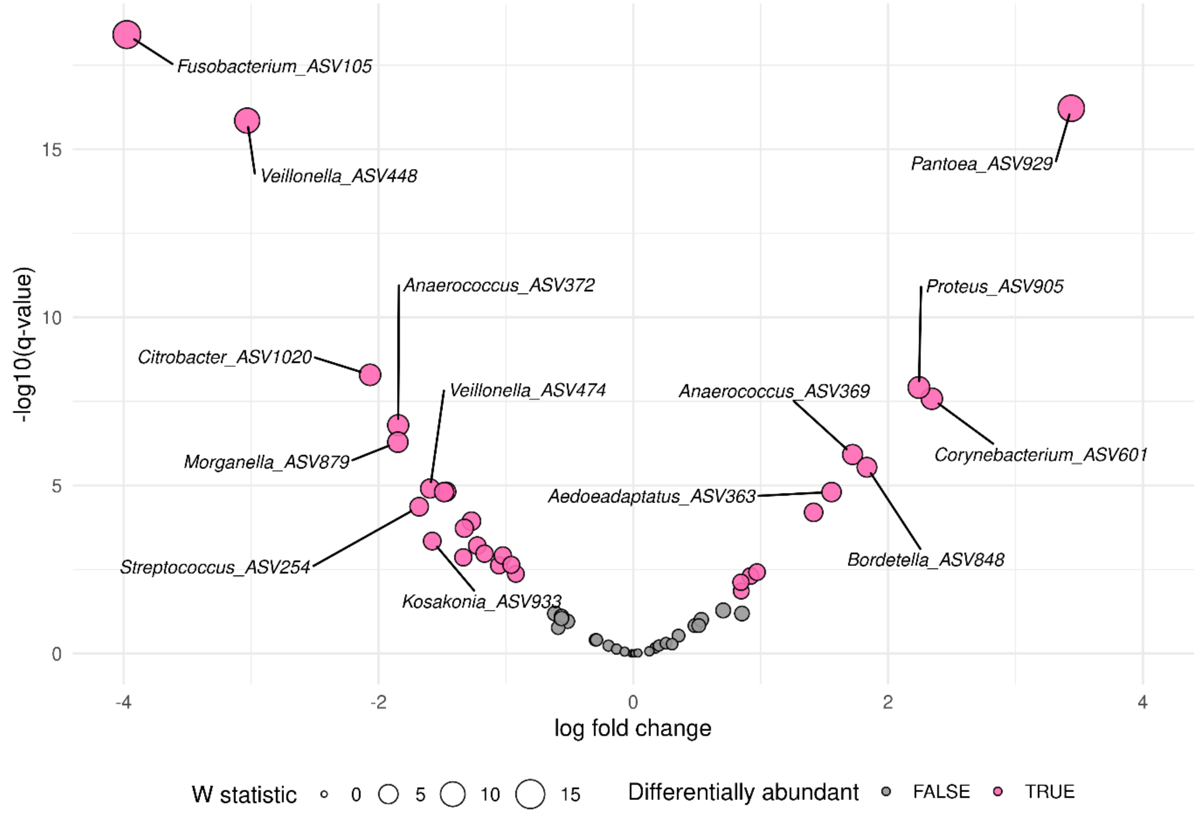

Supplementary Fig. S7. Differentially abundant microbial feature identified by Analysis of Compositions of Microbiomes with Bias Correction 2 (ANCOM-BC2). The volcano plot illustrates the differentially abundant ASVs between the AB and SB groups. The x-axis represents the log fold change, while the y-axis shows the q-values (adjusted p-values). A negative log fold change indicates higher abundance in the AB group compared to the SB group, whereas a positive log fold change signifies higher abundance in the SB group compared to the AB group. The size of the points corresponds to the W-statistic, reflecting the strength of the ANCOM test. Points are color-coded to indicate whether ASVs are differentially abundant (TRUE) or not (FALSE) based on the analysis. ASVs with log fold change more or less than 1.5 are labelled with their respective genera.

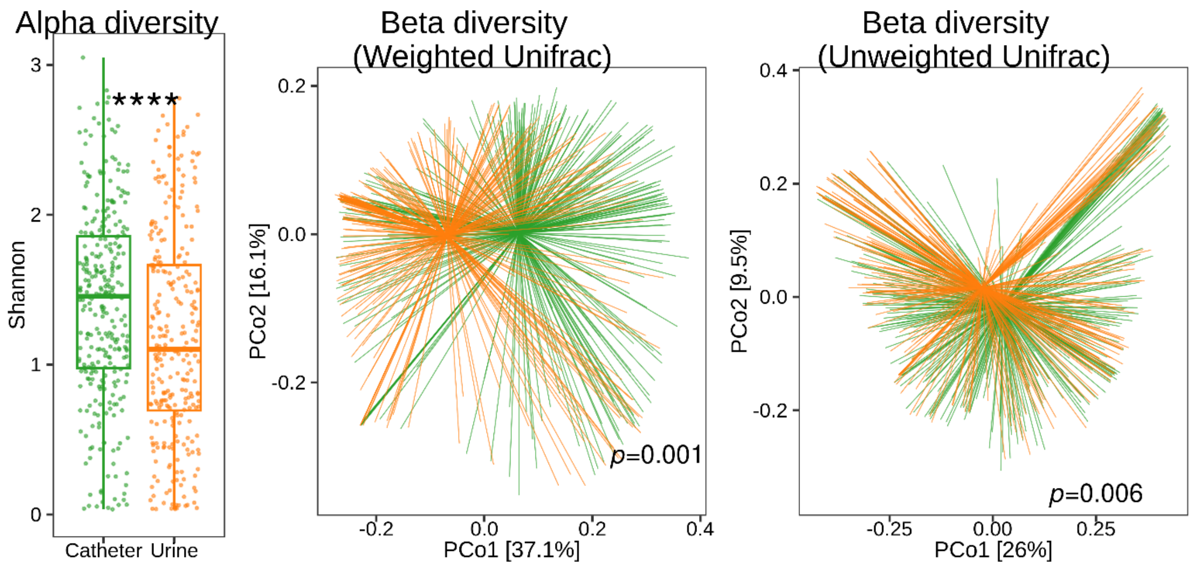

Supplementary Fig. S8. Differences in microbiota community composition between catheter and urine bacteriuria samples. Alpha diversity (left panel) measured by the Shannon index of catheter (green) and urine (orange) samples. Each data point represents an individual sample. Statistical analysis was performed using linear mixed effects model and significance is indicated by, **** $P < 0.0001$ between groups. Principal coordinates analyses (PCoA) of beta-diversity between group based on weighted (middle panel) and unweighted (right panel) unifrac distance matrices are shown with centroid and each line represents an individual sample. Statistical significance was determined by permutational ANOVA (PERMANOVA) with 999 permutations between group and pairwise p-values are indicated inside of the plot.

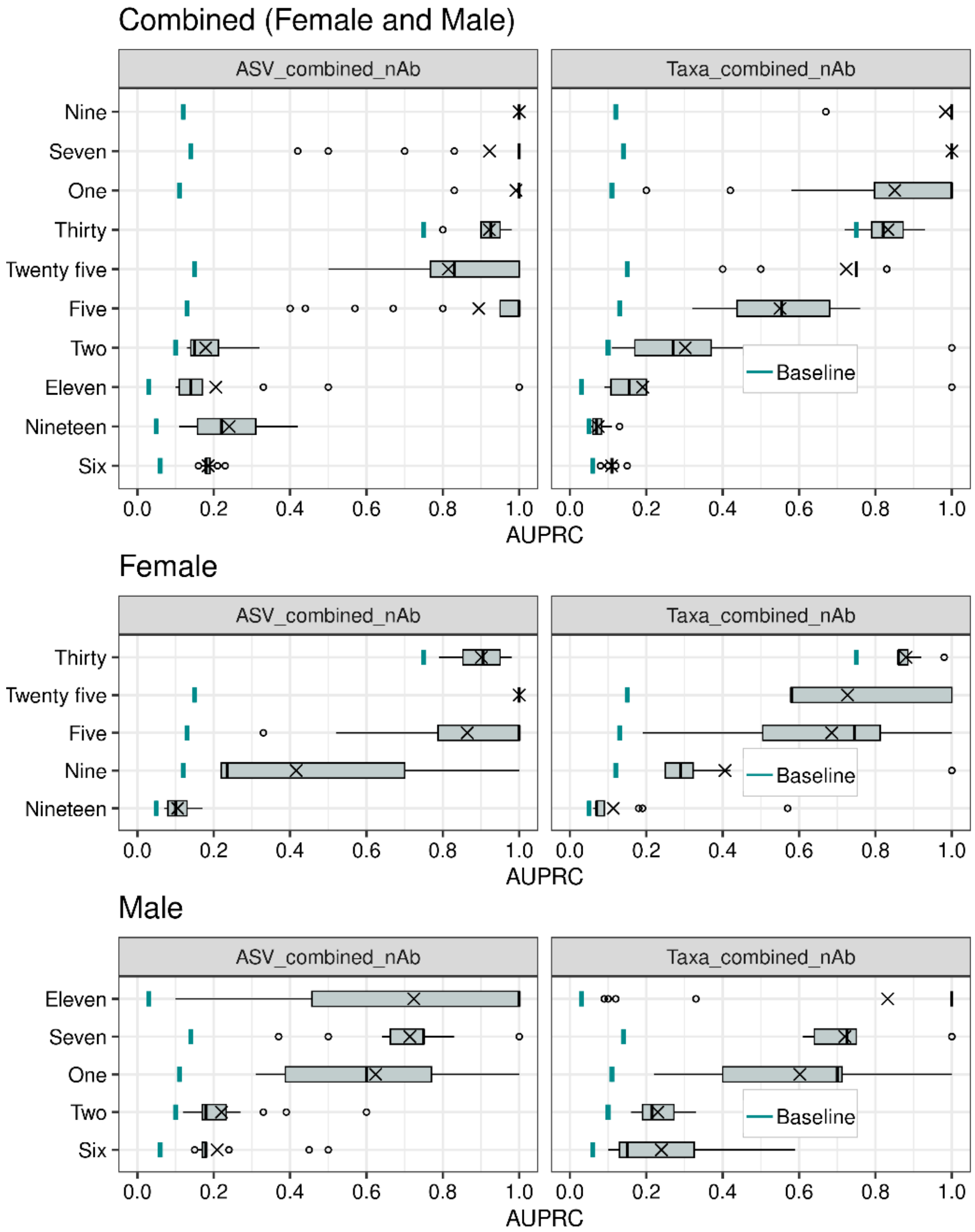

Supplementary Fig. S9. Predictive performance of machine learning models based on leave-one-out-participant type of cross validation using AUPRC scores. The boxplots show performance of ML models using AUPRC on leave-one-out-participant type of cross validation on combined (male and female), female, and male participants. The participants were arranged in descending order from top to bottom based on the mean AUPRC values. The mean depicted as a cross. The median depicted as centre line in the box, edges depict inter-quartiles, and whiskers as distribution of the data (1.5 times of the quartiles). Outliers are shown as points. The baseline chances of AUPRC depicted as vertical solid 'dark-cyan' line underneath of the boxplots for each dataset. The baseline performance for AUPRC was calculated as the fraction of the samples in the minority class (SB) over the total number of samples in the test set.

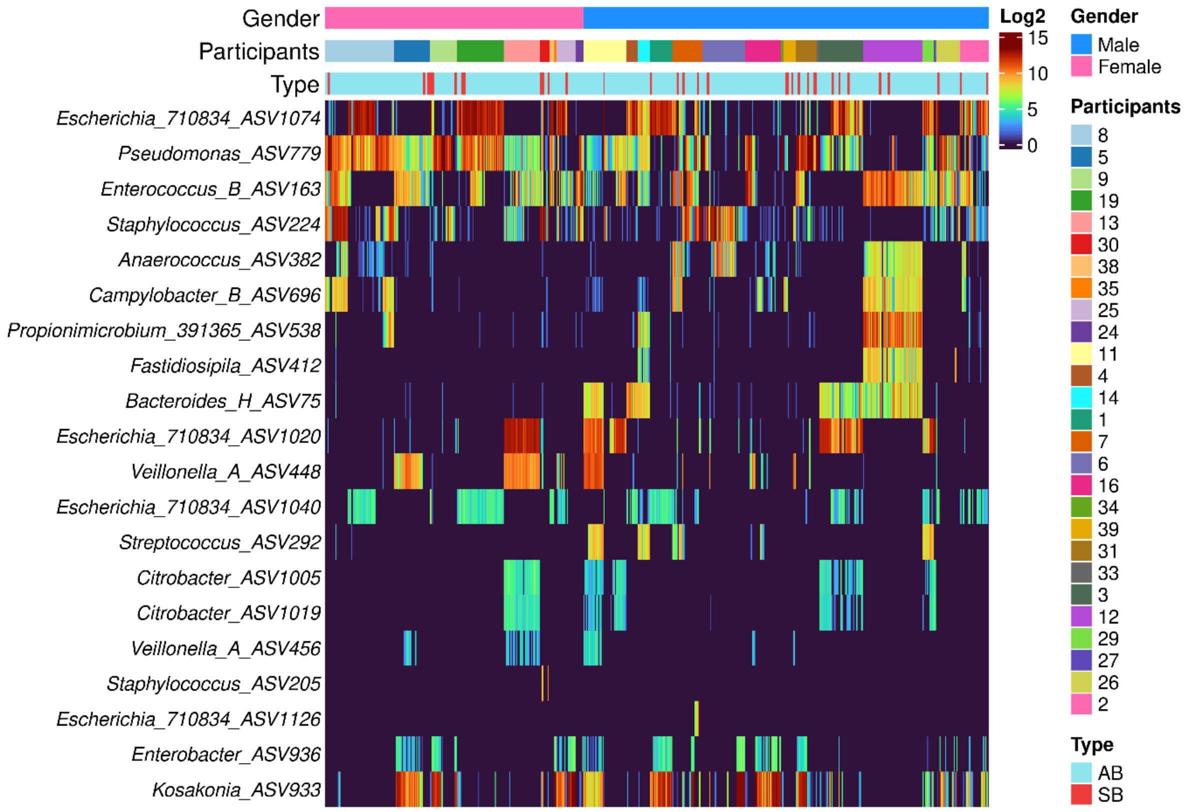

Supplementary Fig. S10. Heatmap of microbial composition with the top 20 most important ASVs across analysed samples. The log2 transformed ASVs counts were used to generate the heatmap (replacing zero's with 1) using MicroViz package in R. Rows are annotated with relevant ASV number and genus level annotations. The bars above the heatmap depicting samples with relevant variable as indicated in the legends on the left side.

Supplementary Tables

Supplementary Table S1. Participants information and samples analysed.

Participant	Received	Analysed	18-months completed	Age	Gender
1	10	10	Deceased	65	Male
2	15	15	Yes	73	Male
3	20	20	Yes	74	Male
4	5	5	Deceased	71	Male
5	17	17	Yes	72	Female
6	20	20	Yes	66	Male
7	22	15	Yes	51	Male
8	32	32	Yes	35	Female
9	15	13	Yes	23	Female
10	Interviewed but excluded because of the criteria did not met.				
11	24	20	Yes	60	Male
12	31	31	Yes	57	Male
13	19	16	Yes	51	Female
14	6	6	Deceased	48	Male
15	Interviewed but excluded because of the criteria did not met.				
16	21	17	Yes	71	Male
17	Interviewed but excluded because of the criteria did not met.				
18	Interviewed but excluded because of the criteria did not met.				
19	26	21	Yes	44	Female
20	Interviewed but excluded because of the criteria did not met.				
21	Interviewed but excluded because of the criteria did not met.				
22	Interviewed but excluded because of the criteria did not met.				
23	Interviewed but excluded because of the criteria did not met.				
24	8	5	No	61	Female
25	16	9	No	58	Female
26	13	11	No	67	Male
27	1	1	withdrawn	50	Male
28	Interviewed but excluded because of the criteria did not met.				
29	7	5	No	56	Male
30	6	5	No	31	Female
31	14	10	No	38	Male
32	Interviewed but excluded because of the criteria did not met.				
33	7	1	No	58	Male
34	2	1	No	58	Male
35	5	1	No	71	Female
36	Interviewed but excluded because of the criteria did not met.				
37	Interviewed but excluded because of the criteria did not met.				
38	4	2	No	63	Female
39	13	6	No	44	Male

Supplementary Table S2. Machine learning models.

ID	Name	Reference
lr	Logistic Regression	sklearn.linear_model._logistic.LogisticRegression
knn	K Neighbors Classifier	sklearn.neighbors._classification.KNeighborsClassifier
nb	Naive Bayes	sklearn.naive_bayes.GaussianNB
dt	Decision Tree Classifier	sklearn.tree._classes.DecisionTreeClassifier
svm	SVM - Linear Kernel	sklearn.linear_model._stochastic_gradient.SGDClassifier
rbfsvm	SVM - Radial Kernel	sklearn.svm._classes.SVC
gpc	Gaussian Process Classifier	sklearn.gaussian_process._gpc.GaussianProcessClassifier
mlp	MLP Classifier	sklearn.neural_network._multilayer_perceptron.MLPClassifier
ridge	Ridge Classifier	sklearn.linear_model._ridge.RidgeClassifier
rf	Random Forest Classifier	sklearn.ensemble._forest.RandomForestClassifier
qda	Quadratic Discriminant Analysis	sklearn.discriminant_analysis.QuadraticDiscriminantAnalysis
ada	Ada Boost Classifier	sklearn.ensemble._weight_boosting.AdaBoostClassifier
gbc	Gradient Boosting Classifier	sklearn.ensemble._gb.GradientBoostingClassifier
lda	Linear Discriminant Analysis	sklearn.discriminant_analysis.LinearDiscriminantAnalysis
et	Extra Trees Classifier	sklearn.ensemble._forest.ExtraTreesClassifier
xgboost	Extreme Gradient Boosting	xgboost.sklearn.XGBClassifier
lightgbm	Light Gradient Boosting Machine	lightgbm.sklearn.LGBMClassifier
catboost	CatBoost Classifier	catboost.core.CatBoostClassifier
dummy	Dummy Classifier	sklearn.dummy.DummyClassifier

Supplementary Table S3. PERMANOVA summary statistics in participants with at least one symptomatic bacteriuria event.

Participants	Measure	F	R2	Pvalue	Significance
1	unwei_unifrac	1.89557097	0.09527603	0.187	
2	unwei_unifrac	1.08084474	0.04309443	0.329	
3	unwei_unifrac	1.31598039	0.03434547	0.269	
5	unwei_unifrac	4.78299903	0.13750968	0.002	**
6	unwei_unifrac	1.62658661	0.04322971	0.13	
7	unwei_unifrac	1.40097714	0.05306535	0.192	
8	unwei_unifrac	1.69171952	0.02787398	0.088	
9	unwei_unifrac	2.3193471	0.09537045	0.05	*
11	unwei_unifrac	1.05348143	0.02843138	0.355	
12	unwei_unifrac	2.32101966	0.04352917	0.065	
19	unwei_unifrac	2.1433536	0.05085864	0.053	
25	unwei_unifrac	1.68368449	0.10091803	0.135	
26	unwei_unifrac	6.3102131	0.24931489	0.009	**
30	unwei_unifrac	0.74375695	0.09604601	0.729	
31	unwei_unifrac	1.35055356	0.07359743	0.219	
39	unwei_unifrac	1.19922045	0.11757962	0.299	
1	wei_unifrac	0.24575563	0.01346919	0.701	
2	wei_unifrac	0.62641781	0.02543682	0.66	
3	wei_unifrac	1.22590169	0.03206992	0.291	
5	wei_unifrac	3.74948072	0.11109743	0.011	*
6	wei_unifrac	0.23451542	0.00647216	0.907	
7	wei_unifrac	2.6284061	0.09513419	0.027	*
8	wei_unifrac	3.02306764	0.04874102	0.008	**
9	wei_unifrac	5.96435925	0.21328432	0.003	**
11	wei_unifrac	1.14817884	0.03090808	0.259	
12	wei_unifrac	0.55851575	0.01083266	0.613	
19	wei_unifrac	0.40189597	0.00994745	0.701	
25	wei_unifrac	1.68442532	0.10095795	0.069	
26	wei_unifrac	1.49529062	0.07295777	0.194	
30	wei_unifrac	1.10565333	0.13640521	0.431	
31	wei_unifrac	0.9270493	0.05171232	0.361	
39	wei_unifrac	0.51339439	0.05396543	0.702	

Supplementary Table S4. Differentially abundant microbial feature identified by Analysis of Compositions of Microbiomes with Bias Correction 2 (ANCOM-BC2).

ASV	lcf	W	p_value	q_value	Diff_ab
Prevotella_ASV25	0.84719435	2.71397758	0.00754304	0.01382891	TRUE
Prevotella_ASV26	0.92030753	3.14616477	0.00251051	0.00493136	TRUE
Bacteroides_H_ASV75	-1.46796221	-4.83697393	3.3301E-06	1.5263E-05	TRUE
Fusobacterium_C_ASV97	-1.27014871	-4.40078906	3.5662E-05	0.00011538	TRUE
Fusobacterium_C_ASV105	-3.97519284	-13.7283768	7.0487E-21	3.8768E-19	TRUE
Lactobacillus_ASV130	0.17235294	0.54090648	0.58985157	0.67587159	FALSE
Enterococcus_B_ASV163	-1.22481456	-3.73446824	0.00022431	0.00061686	TRUE
Staphylococcus_ASV220	0.4841518	1.641083	0.10475582	0.14773257	FALSE
Staphylococcus_ASV224	0.85286286	2.08595125	0.0380094	0.06438959	FALSE
Streptococcus_ASV254	-1.68108632	-4.50212723	1.1716E-05	4.2958E-05	TRUE
Streptococcus_ASV262	-0.51614782	-1.81370292	0.07407184	0.11010678	FALSE
Streptococcus_ASV265	-0.1949375	-0.68292936	0.49657645	0.5811001	FALSE
Streptococcus_ASV283	-1.05422674	-3.34113057	0.00108957	0.00239705	TRUE
Streptococcus_ASV290	0.20067448	0.70344032	0.48404999	0.57875542	FALSE
Peptostreptococcus_ASV306	-0.01228925	-0.04288158	0.96591978	0.9891274	FALSE
Parvimonas_ASV319	-1.02399999	-3.60083211	0.00048428	0.0012107	TRUE
Finegoldia_ASV333	1.41534705	4.39439589	1.8356E-05	6.3098E-05	TRUE
Peptoniphilus_A_ASV344	0.70569594	2.19307097	0.02935009	0.05207275	FALSE
Peptoniphilus_C_ASV363	1.55619786	5.10510514	3.7117E-06	1.5704E-05	TRUE
Anaerococcus_ASV369	1.72133387	5.62662824	1.9682E-07	1.2028E-06	TRUE
Anaerococcus_ASV372	-1.84570318	-6.49537819	2.0522E-08	1.6124E-07	TRUE
Anaerococcus_ASV382	-0.6153212	-2.08759366	0.03863375	0.06438959	FALSE
Fastidiosipila_ASV412	0.53409529	1.883274	0.06413867	0.09798963	FALSE
Veillonella_A_ASV448	-3.03092926	-10.5299132	7.6612E-18	1.4045E-16	TRUE
Veillonella_A_ASV453	-1.16784836	-3.5949287	0.00040736	0.00106689	TRUE
Veillonella_A_ASV458	-0.56345992	-1.99351867	0.04901304	0.07928581	FALSE
Veillonella_A_ASV474	-1.59504731	-4.91798619	2.4649E-06	1.2324E-05	TRUE
Veillonella_A_ASV482	-0.92178123	-3.20009015	0.00210052	0.00427883	TRUE
Propionimicrobium_391365_ASV538	-1.32624776	-4.20611579	6.1261E-05	0.00018719	TRUE
Varibaculum_ASV572	-0.13159983	-0.4484865	0.6550782	0.73529186	FALSE
Pauljensenia_ASV578	-0.2976613	-1.04837314	0.29867147	0.39105544	FALSE
Corynebacterium_ASV599	0.51364485	1.64668036	0.10185079	0.14741562	FALSE
Corynebacterium_ASV601	2.34315352	6.77530107	2.8621E-09	2.6236E-08	TRUE
Actinobaculum_ASV610	0	0	1	1	FALSE
Actinotignum_ASV621	-0.58974012	-1.54345859	0.12375644	0.1701651	FALSE
Actinotignum_ASV637	-0.06802196	-0.23821956	0.81199437	0.8756802	FALSE
Actinotignum_ASV658	0.01025733	0.03622871	0.97114326	0.9891274	FALSE
Trueperella_ASV664	-1.48511675	-5.08467175	4.0001E-06	1.5715E-05	TRUE
Campylobacter_B_ASV696	0.35453846	1.2348554	0.21893817	0.29369754	FALSE
Pseudomonas_ASV779	0.1260071	0.28116492	0.77872749	0.85660024	FALSE
Pseudomonas_ASV780	0.97105291	3.17076212	0.0017874	0.00378104	TRUE
Serratia_D_727363_ASV821	-1.33423434	-3.54208072	0.00057452	0.00137386	TRUE
Achromobacter_ASV848	1.83387599	5.35821961	5.2326E-07	2.8779E-06	TRUE
Morganella_ASV879	-1.84827728	-5.91795315	7.4958E-08	5.1533E-07	TRUE
Proteus_ASV905	2.24199058	7.07615568	1.1066E-09	1.2172E-08	TRUE
Citrobacter_ASV917	-0.56357741	-1.92839026	0.05774988	0.09074981	FALSE
Pantoea_ASV929	3.43771083	12.0219095	2.1924E-18	6.0291E-17	TRUE
Kosakonia_ASV933	-1.57823145	-3.84868733	0.00015583	0.00045108	TRUE
Enterobacter_ASV936	0.8448763	2.93653028	0.00398554	0.00755879	TRUE
Citrobacter_ASV1005	-0.28854716	-1.02988666	0.30573425	0.39105544	FALSE
Citrobacter_ASV1019	-0.9579149	-3.40637922	0.00100291	0.00229834	TRUE
Escherichia_710834_ASV1020	-2.06615658	-6.76343892	3.725E-10	5.1219E-09	TRUE
Escherichia_710834_ASV1040	0.03553163	0.11468984	0.90882711	0.96125945	FALSE
Escherichia_710834_ASV1067	0.25665164	0.85345922	0.39462717	0.49328397	FALSE
Escherichia_710834_ASV1074	0.30442881	0.79181207	0.42925216	0.52464153	FALSE

Supplementary Table S5 Performance of machine learning models during cross-validation.

		Cross-validation set											
		Without antibiotic treated samples						With antibiotic treated samples					
		Combined		Catheter		Urine		Combined		Catheter		Urine	
		ASV	Taxa	ASV	Taxa	ASV	Taxa	ASV	Taxa	ASV	Taxa	ASV	Taxa
AUROC	min	0.91	0.78	0.83	0.72	0.87	0.72	0.82	0.72	0.73	0.67	0.68	0.67
AUROC	max	0.98	0.91	0.98	0.97	0.98	0.87	0.89	0.84	0.91	0.82	0.84	0.8
AUROC	q25	0.93	0.86	0.88	0.82	0.92	0.76	0.83	0.77	0.81	0.72	0.73	0.71
AUROC	q75	0.96	0.88	0.93	0.87	0.95	0.83	0.86	0.81	0.86	0.77	0.78	0.75
AUROC	mean	0.95	0.87	0.9	0.84	0.94	0.8	0.85	0.79	0.83	0.74	0.76	0.73
AUROC	std	0.02	0.03	0.04	0.06	0.03	0.04	0.02	0.03	0.05	0.04	0.04	0.04
Precision	min	0.94	0.94	0.93	0.91	0.94	0.91	0.9	0.85	0.1	0.85	0.82	0.84
Precision	max	0.97	0.96	0.97	0.96	0.97	0.95	0.91	0.9	0.91	0.89	0.9	0.89
Precision	q25	0.95	0.94	0.94	0.93	0.95	0.93	0.91	0.88	0.89	0.86	0.88	0.87
Precision	q75	0.96	0.95	0.95	0.95	0.96	0.94	0.91	0.9	0.9	0.88	0.89	0.88
Precision	mean	0.95	0.95	0.95	0.94	0.95	0.93	0.91	0.89	0.84	0.87	0.88	0.87
Precision	std	0.01	0.01	0.01	0.01	0.01	0.01	0	0.01	0.19	0.01	0.02	0.01
Recall	min	0.76	0.33	0.59	0.61	0.47	0.45	0.39	0.4	0.11	0.5	0.17	0.48
Recall	max	0.97	0.96	0.94	0.93	0.9	0.95	0.64	0.86	0.86	0.84	0.7	0.84
Recall	q25	0.83	0.58	0.77	0.7	0.74	0.62	0.42	0.53	0.68	0.61	0.43	0.55
Recall	q75	0.91	0.78	0.83	0.88	0.84	0.86	0.6	0.66	0.77	0.71	0.59	0.62
Recall	mean	0.87	0.68	0.8	0.79	0.78	0.75	0.52	0.6	0.68	0.66	0.51	0.6
Recall	std	0.06	0.18	0.07	0.11	0.1	0.15	0.1	0.12	0.2	0.09	0.12	0.09
F1	min	0.82	0.43	0.69	0.71	0.58	0.56	0.45	0.47	0.04	0.58	0.14	0.56
F1	max	0.96	0.95	0.94	0.93	0.92	0.94	0.71	0.85	0.87	0.85	0.76	0.84
F1	q25	0.87	0.68	0.83	0.78	0.81	0.71	0.5	0.6	0.74	0.68	0.5	0.62
F1	q75	0.93	0.83	0.87	0.9	0.88	0.89	0.67	0.72	0.81	0.76	0.67	0.69
F1	mean	0.9	0.75	0.85	0.84	0.83	0.81	0.59	0.67	0.71	0.71	0.57	0.67
F1	std	0.04	0.15	0.05	0.07	0.08	0.11	0.1	0.1	0.22	0.07	0.14	0.07
Accuracy	min	0.76	0.33	0.59	0.61	0.47	0.45	0.39	0.4	0.11	0.5	0.17	0.48
Accuracy	max	0.97	0.96	0.94	0.93	0.9	0.95	0.64	0.86	0.86	0.84	0.7	0.84
Accuracy	q25	0.83	0.58	0.77	0.7	0.74	0.62	0.42	0.53	0.68	0.61	0.43	0.55
Accuracy	q75	0.91	0.78	0.83	0.88	0.84	0.86	0.6	0.66	0.77	0.71	0.59	0.62
Accuracy	mean	0.87	0.68	0.8	0.79	0.78	0.75	0.52	0.6	0.68	0.66	0.51	0.6
Accuracy	std	0.06	0.18	0.07	0.11	0.1	0.15	0.1	0.12	0.2	0.09	0.12	0.09
Balanced accuracy	min	0.72	0.65	0.63	0.58	0.72	0.59	0.66	0.61	0.5	0.61	0.54	0.58
Balanced accuracy	max	0.89	0.82	0.89	0.88	0.93	0.82	0.77	0.78	0.82	0.72	0.75	0.72
Balanced accuracy	q25	0.83	0.7	0.78	0.66	0.82	0.66	0.67	0.67	0.74	0.64	0.63	0.63
Balanced accuracy	q75	0.86	0.79	0.83	0.78	0.88	0.75	0.75	0.72	0.78	0.7	0.7	0.69
Balanced accuracy	mean	0.84	0.75	0.8	0.72	0.84	0.71	0.72	0.69	0.74	0.67	0.66	0.66
Balanced accuracy	std	0.04	0.05	0.07	0.09	0.05	0.07	0.04	0.04	0.08	0.04	0.05	0.04

Supplementary Table S6. Performance of machine learning models on held-out set.

		Held-out set											
		Without antibiotic treated samples						With antibiotic treated samples					
		Combined		Catheter		Urine		Combined		Catheter		Urine	
		ASV	Taxa	ASV	Taxa	ASV	Taxa	ASV	Taxa	ASV	Taxa	ASV	Taxa
AUROC	min	0.85	0.69	0.7	0.68	0.85	0.57	0.69	0.55	0.54	0.53	0.47	0.48
AUROC	max	1	0.99	0.99	0.96	1	1	0.93	0.86	0.96	0.85	0.84	0.8
AUROC	q25	0.93	0.78	0.85	0.76	0.93	0.69	0.81	0.66	0.74	0.61	0.6	0.58
AUROC	q75	0.98	0.88	0.96	0.87	0.98	0.89	0.88	0.76	0.9	0.69	0.74	0.75
AUROC	mean	0.95	0.83	0.88	0.81	0.95	0.79	0.83	0.71	0.82	0.66	0.67	0.65
AUROC	std	0.04	0.08	0.1	0.08	0.03	0.13	0.07	0.08	0.11	0.09	0.11	0.11
Precision	min	0.93	0.91	0.92	0.87	0.94	0.89	0.87	0.81	0.86	0.8	0.81	0.78
Precision	max	0.97	0.96	0.96	0.98	0.98	0.97	0.92	0.92	0.93	0.92	0.92	0.92
Precision	q25	0.95	0.93	0.94	0.91	0.96	0.92	0.91	0.86	0.9	0.84	0.86	0.85
Precision	q75	0.96	0.95	0.95	0.95	0.97	0.96	0.91	0.89	0.93	0.88	0.91	0.88
Precision	mean	0.96	0.94	0.95	0.92	0.96	0.94	0.91	0.88	0.91	0.86	0.87	0.86
Precision	std	0.01	0.01	0.01	0.03	0.01	0.02	0.01	0.03	0.02	0.03	0.03	0.04
Recall	min	0.73	0.32	0.63	0.65	0.54	0.5	0.35	0.36	0.11	0.44	0.25	0.39
Recall	max	0.97	0.93	0.96	0.98	0.96	0.93	0.68	0.88	0.9	0.82	0.68	0.82
Recall	q25	0.82	0.55	0.77	0.71	0.73	0.6	0.43	0.53	0.65	0.53	0.39	0.53
Recall	q75	0.92	0.75	0.92	0.88	0.88	0.87	0.62	0.62	0.78	0.73	0.58	0.63
Recall	mean	0.87	0.67	0.82	0.8	0.8	0.76	0.53	0.58	0.67	0.63	0.48	0.58
Recall	std	0.06	0.16	0.09	0.1	0.1	0.14	0.11	0.11	0.21	0.12	0.11	0.1
F1	min	0.8	0.42	0.73	0.74	0.66	0.62	0.42	0.43	0.05	0.53	0.27	0.46
F1	max	0.97	0.92	0.95	0.98	0.96	0.94	0.74	0.87	0.91	0.85	0.75	0.84
F1	q25	0.87	0.66	0.83	0.79	0.81	0.72	0.51	0.61	0.72	0.63	0.46	0.61
F1	q75	0.93	0.82	0.92	0.9	0.91	0.9	0.7	0.69	0.82	0.78	0.66	0.7
F1	mean	0.9	0.75	0.86	0.84	0.85	0.82	0.61	0.66	0.71	0.7	0.55	0.65
F1	std	0.04	0.13	0.06	0.07	0.07	0.1	0.11	0.09	0.23	0.09	0.12	0.09
Accuracy	min	0.73	0.32	0.63	0.65	0.54	0.5	0.35	0.36	0.11	0.44	0.25	0.39
Accuracy	max	0.97	0.93	0.96	0.98	0.96	0.93	0.68	0.88	0.9	0.82	0.68	0.82
Accuracy	q25	0.82	0.55	0.77	0.71	0.73	0.6	0.43	0.53	0.65	0.53	0.39	0.53
Accuracy	q75	0.92	0.75	0.92	0.88	0.88	0.87	0.62	0.62	0.78	0.73	0.58	0.63
Accuracy	mean	0.87	0.67	0.82	0.8	0.8	0.76	0.53	0.58	0.67	0.63	0.48	0.58
Accuracy	std	0.06	0.16	0.09	0.1	0.1	0.14	0.11	0.11	0.21	0.12	0.11	0.1
Balanced_accuracy	min	0.7	0.57	0.64	0.45	0.67	0.32	0.64	0.46	0.51	0.42	0.49	0.44
Balanced_accuracy	max	0.97	0.86	0.96	0.88	0.98	0.91	0.82	0.75	0.88	0.75	0.78	0.81
Balanced_accuracy	q25	0.81	0.65	0.73	0.63	0.85	0.53	0.68	0.62	0.67	0.56	0.58	0.58
Balanced_accuracy	q75	0.92	0.75	0.89	0.82	0.93	0.78	0.77	0.7	0.83	0.67	0.67	0.68
Balanced_accuracy	mean	0.86	0.71	0.82	0.71	0.88	0.66	0.72	0.65	0.74	0.61	0.63	0.63
Balanced_accuracy	std	0.08	0.08	0.09	0.13	0.07	0.16	0.05	0.07	0.11	0.09	0.07	0.1
AUPRC	min	0.37	0.13	0.13	0.16	0.18	0.07	0.21	0.14	0.14	0.12	0.11	0.11
AUPRC	max	1	0.85	0.87	0.74	1	1	0.62	0.51	0.82	0.32	0.53	0.54
AUPRC	q25	0.6	0.26	0.24	0.2	0.35	0.14	0.34	0.19	0.29	0.14	0.16	0.14
AUPRC	q75	0.84	0.48	0.6	0.47	0.58	0.39	0.53	0.33	0.62	0.22	0.3	0.33
AUPRC	mean	0.7	0.39	0.45	0.36	0.49	0.3	0.43	0.27	0.48	0.19	0.25	0.25
AUPRC	std	0.18	0.2	0.22	0.19	0.2	0.25	0.12	0.1	0.21	0.07	0.11	0.13
Baseline		0.05	0.05	0.06	0.06	0.04	0.04	0.1	0.1	0.1	0.1	0.11	0.11

Supplementary Table S7. Performance of machine learning models using leave-one-participant out type of cross validation across female and male participants.

		Accuracy		AUROC		Recall		Precision		F1		Balanced accuracy		AUPRC		Baseline AUPRC
		mean	std	mean	std	mean	std	mean	std	mean	std	mean	std	mean	std	mean
	Participant	Combine (Female and Male)														
ASV	Eleven	0.85	0.09	0.84	0.07	0.85	0.09	0.95	0.01	0.89	0.06	0.51	0.14	0.21	0.21	0.03
	Five	0.92	0.08	0.98	0.04	0.92	0.08	0.88	0.12	0.89	0.09	0.77	0.24	0.89	0.2	0.13
	Nine	0.83	0.05	1	0	0.83	0.05	0.93	0.02	0.85	0.04	0.9	0.03	1	0	0.12
	Nineteen	0.43	0.26	0.82	0.11	0.43	0.26	0.95	0.03	0.5	0.25	0.67	0.15	0.24	0.1	0.05
	One	0.89	0.09	1	0	0.89	0.09	0.93	0.07	0.89	0.07	0.84	0.17	0.99	0.04	0.11
	Seven	0.95	0.06	0.98	0.04	0.95	0.06	0.96	0.07	0.95	0.07	0.89	0.15	0.92	0.18	0.14
	Six	0.79	0.01	0.81	0.02	0.79	0.01	0.94	0.02	0.85	0.01	0.82	0.14	0.19	0.02	0.06
	Thirty	0.43	0.15	0.75	0.1	0.43	0.15	0.63	0.34	0.38	0.2	0.6	0.07	0.92	0.04	0.75
	Twenty five	0.81	0.06	0.95	0.04	0.81	0.06	0.92	0.02	0.84	0.05	0.89	0.04	0.81	0.17	0.15
	Two	0.74	0.13	0.6	0.1	0.74	0.13	0.81	0.04	0.77	0.08	0.44	0.13	0.18	0.05	0.1
Taxa	Eleven	0.78	0.12	0.84	0.07	0.78	0.12	0.96	0.02	0.85	0.08	0.6	0.2	0.19	0.2	0.03
	Five	0.86	0.04	0.9	0.06	0.86	0.04	0.77	0.05	0.81	0.03	0.53	0.11	0.55	0.14	0.13
	Nine	0.94	0.15	1	0.02	0.94	0.15	0.98	0.03	0.95	0.14	0.97	0.09	0.98	0.07	0.12
	Nineteen	0.21	0.14	0.47	0.12	0.21	0.14	0.69	0.41	0.26	0.2	0.54	0.12	0.07	0.02	0.05
	One	0.87	0.14	0.95	0.1	0.87	0.14	0.87	0.08	0.85	0.14	0.65	0.17	0.85	0.23	0.11
	Seven	0.98	0.04	1	0	0.98	0.04	0.97	0.06	0.97	0.06	0.91	0.15	1	0	0.14
	Six	0.75	0.07	0.64	0.05	0.75	0.07	0.88	0.01	0.81	0.04	0.43	0.09	0.11	0.01	0.06
	Thirty	0.29	0.08	0.57	0.11	0.29	0.08	0.24	0.32	0.18	0.14	0.52	0.05	0.83	0.06	0.75
	Twenty five	0.82	0.05	0.91	0.03	0.82	0.05	0.88	0.06	0.83	0.04	0.81	0.13	0.72	0.12	0.15
	Two	0.72	0.17	0.71	0.2	0.72	0.17	0.85	0.05	0.75	0.14	0.57	0.13	0.3	0.2	0.1
Female																
ASV	Five	0.87	0.01	0.97	0.04	0.87	0.01	0.76	0.04	0.81	0.02	0.51	0.03	0.86	0.2	0.13
	Nine	0.61	0.11	0.81	0.09	0.61	0.11	0.91	0.01	0.67	0.09	0.76	0.01	0.42	0.27	0.12
	Nineteen	0.61	0.18	0.63	0.14	0.61	0.18	0.93	0.02	0.7	0.18	0.64	0.08	0.11	0.03	0.05
	Thirty	0.31	0.06	0.75	0.12	0.31	0.06	0.44	0.39	0.21	0.12	0.54	0.04	0.9	0.07	0.75
	Twenty five	0.97	0.05	1	0	0.97	0.05	0.97	0.07	0.96	0.06	0.95	0.12	1	0	0.15
Taxa	Five	0.89	0.04	0.85	0.11	0.89	0.04	0.86	0.09	0.86	0.06	0.67	0.16	0.69	0.22	0.13
	Nine	0.72	0.13	0.84	0.07	0.72	0.13	0.91	0.06	0.76	0.11	0.8	0.14	0.41	0.27	0.12
	Nineteen	0.63	0.14	0.59	0.11	0.63	0.14	0.92	0.01	0.72	0.15	0.59	0.05	0.11	0.12	0.05
	Thirty	0.39	0.06	0.69	0.09	0.39	0.06	0.79	0.17	0.34	0.09	0.59	0.04	0.88	0.03	0.75
	Twenty five	0.91	0.07	0.96	0.03	0.91	0.07	0.92	0.07	0.91	0.07	0.85	0.15	0.73	0.21	0.15
Male																
ASV	Eleven	0.74	0.25	0.96	0.08	0.74	0.25	0.97	0.01	0.8	0.19	0.77	0.18	0.72	0.36	0.03
	One	0.33	0.16	0.9	0.07	0.33	0.16	0.9	0.03	0.35	0.17	0.6	0.06	0.62	0.25	0.11
	Seven	0.88	0.04	0.89	0.06	0.88	0.04	0.88	0.05	0.87	0.04	0.71	0.05	0.71	0.13	0.14
	Six	0.94	0	0.81	0.05	0.94	0	0.89	0	0.92	0	0.5	0	0.21	0.09	0.06
	Two	0.6	0.15	0.63	0.1	0.6	0.15	0.86	0.04	0.67	0.13	0.6	0.1	0.22	0.11	0.1
Taxa	Eleven	0.97	0.06	0.96	0.09	0.97	0.06	0.98	0.02	0.97	0.04	0.84	0.23	0.83	0.35	0.03
	One	0.67	0.12	0.9	0.05	0.67	0.12	0.92	0.01	0.73	0.1	0.82	0.07	0.6	0.21	0.11
	Seven	0.85	0.12	0.87	0.07	0.85	0.12	0.89	0.06	0.86	0.1	0.71	0.07	0.72	0.11	0.14
	Six	0.87	0.08	0.75	0.13	0.87	0.08	0.89	0.02	0.88	0.04	0.51	0.13	0.24	0.16	0.06
	Two	0.83	0.05	0.67	0.07	0.83	0.05	0.82	0.02	0.82	0.02	0.49	0.07	0.23	0.05	0.1

Supplementary Table S8. Top 20 ASVs for model prediction and their exact nucleotide sequences.

ASV_ no	Genus	Importance Score	Sequence
1126	*g_Escherichia*	0.80	TACGGAGGGTGCAAGCGTTAATCGGAATTACTGGGCGTAAAGCGCA<br>CGCAGGCGGTTTGTTAAGTCAGATGTGAAATCCCCGGGCTCAACCT<br>GGGAACTGCATCTGATACTGGCAAGCTTGAGTCTCGTAGAGGGGGG<br>TAGAATTCCAGGTGTAGCGGTGAAATGCGTAGAGATCTGGAGGAAT<br>ACCGGTGGCGAAGGCGGCCCCCTGGACGAAGACTGACGCTCAGGTG<br>TGAAAGCGTGGGGAGCAAACAGG
224	*g_Staphylococcus*	0.68	TACGTAGGTGGCAAGCGTTATCCGGAATTATTGGGCGTAAAGCGCG<br>CGTAGGCGGTTTTTTAAGTCTGATGTGAAAGCCCACGGCTCAACCGT<br>GGAGGGTCATTGGAAACTGGAAAACTTGAGTGCAGAAGAGGAAAG<br>TGGAATTCCATGTGTAGCGGTGAAATGCGCAGAGATATGGAGGAAC<br>ACCAGTGGCGAAGGCGACTTTCTGGTCTGTAACTGACGCTGATGTG<br>CGAAAGCGTGGGGATCAAACAGG
1040	*g_Escherichia*	0.63	GTGTGCCAGCCGCGGTAATACGGAGGGTGCAAGCGTTAATCGGAAT<br>TACTGGGCGTAAAGCGCACGCAGGCGGTTTGTTAAGTCAGATGTGA<br>AATCCCCGGGCTCAACCTGGGAACTGCATCTGATACTGGCAAGCTT<br>GAGTCTCGTAGAGGGGGGTAGAATTCCAGGTGTAGCGGTGAAATGC<br>GTAGAGATCTGGAGGAATACCGGTGGCGAAGGCGGCCCCCTGGACG<br>AAGACTGACGCTCAGGTGCGAAAGCGTGGGGAGCAAACAGG
1074	*g_Escherichia*	0.61	TACGGAGGGTGCAAGCGTTAATCGGAATTACTGGGCGTAAAGCGCA<br>CGCAGGCGGTTTGTTAAGTCAGATGTGAAATCCCCGGGCTCAACCT<br>GGGAACTGCATCTGATACTGGCAAGCTTGAGTCTCGTAGAGGGGGG<br>TAGAATTCCAGGTGTAGCGGTGAAATGCGTAGAGATCTGGAGGAAT<br>ACCGGTGGCGAAGGCGGCCCCCTGGACGAAGACTGACGCTCAGGTG<br>CGAAAGCGTGGGGAGCAAACAGG
1020	*g_Citrobacter*	0.42	TACGGAGGGTGCAAGCGTTAATCGGAATTACTGGGCGTAAAGCGCA<br>CGCAGGCGGTCTGTTAAGTCAGATGTGAAATCCCCGGGCTCAACCT<br>GGGAACTGCATCTGATACTGGCAGGCTTGAGTCTCGTAGAGGGGGG<br>TAGAATTCCAGGTGTAGCGGTGAAATGCGTAGAGATCTGGAGGAAT<br>ACCGGTGGCGAAGGCGGCCCCCTGGACGAAGACTGACGCTCAGGTG<br>CGAAAGCGTGGGGAGCAAACAGG
1005	*g_Citrobacter*	0.40	GTGTGCCAGCCGCGGTAATACGGAGGGTGCAAGCGTTAATCGGAAT<br>TACTGGGCGTAAAGCGCACGCAGGCGGTCTGTTAAGTCAGATGTGA<br>AATCCCCGGGCTCAACCTGGGAACTGCATCTGATACTGGCAGGCTT<br>GAGTCTCGTAGAGGGGGGTAGAATTCCAGGTGTAGCGGTGAAATGC<br>GTAGAGATCTGGAGGAATACCGGTGGCGAAGGCGGCCCCCTGGACG<br>AAGACTGACGCTCAGGTGCGAAAGCGTGGGGAGCAAACAGG
1019	*g_Citrobacter*	0.39	GTGTGTCAGCCGCGGTAATACGGAGGGTGCAAGCGTTAATCGGAAT<br>TACTGGGCGTAAAGCGCACGCAGGCGGTCTGTTAAGTCAGATGTGA<br>AATCCCCGGGCTCAACCTGGGAACTGCATCTGATACTGGCAGGCTT<br>GAGTCTCGTAGAGGGGGGTAGAATTCCAGGTGTAGCGGTGAAATGC<br>GTAGAGATCTGGAGGAATACCGGTGGCGAAGGCGGCCCCCTGGACG<br>AAGACTGACGCTCAGGTGCGAAAGCGTGGGGAGCAAACAGG
936	*g_Enterobacter*	0.39	GTGTGCCAGCCGCGGTAATACGGAGGGTGCAAGCGTTAATCGGAAT<br>TACTGGGCGTAAAGCGCACGCAGGCGGTCTGTCAAGTCGGATGTGA<br>AATCCCCGGGCTCAACCTGGGAACTGCATTCGAAACTGGCAGGCTA<br>GAGTCTTGTAGAGGGGGGTAGAATTCCAGGTGTAGCGGTGAAATGC<br>GTAGAGATCTGGAGGAATACCGGTGGCGAAGGCGGCCCCCTGGACA<br>AAGACTGACGCTCAGGTGCGAAAGCGTGGGGAGCAAACAGG
448	*g_Veillonella*	0.37	TACGTAGGTGGCAAGCGTTGTCCGGAATTATTGGGCGTAAAGCGCG<br>CGCAGGCGGATCGGTCAGTCTGTCTTAAAAGTTCGGGGCTTAACCC<br>CGTGATGGGATGGAAACTGCCAATCTAGAGTATCGGAGAGGAAAGT<br>GGAATTCCTAGTGTAGCGGTGAAATGCGTAGATATTAGGAAGAACA<br>CCAGTGGCGAAGGCGACTTTCTGGACGAAAACTGACGCTGAGGCGC<br>GAAAGCCAGGGGAGCGAACGGG
696	*g_Campylobacter*	0.36	TACGGGGGGTGCAAGCGTTACTCGGAATCACTGGGCGTAAAGGACG<br>CGTAGGCGGATTATCAAGTCTTTTGTGAAATCTAGTGGCTTAACCAC<br>TAAACTGCTTAGGAAACTGATAGTCTAGAGTAGGGGAGAGGTAGAT<br>GGAATTCTTGGTGTAGGGGTAAAATCCGTAGAGATCAAGAAGAATA<br>CCCATTGCGAAAGCGATCTGCTGGAACCTAACTGACGCTGAGGCGT<br>GAAAGCGTGGGTAGCAAACAGG
933	*g_Kosakonia*	0.35	TACGGAGGGTGCAAGCGTTAATCGGAATTACTGGGCGTAAAGCGCA<br>CGCAGGCGGTCTGTCAAGTCGGATGTGAAATCCCCGGGCTCAACCT<br>GGGAACTGCATTCGAAACTGGCAGGCTAGAGTCTTGTAGAGGGGGG<br>TAGAATTCCAGGTGTAGCGGTGAAATGCGTAGAGATCTGGAGGAAT<br>ACCGGTGGCGAAGGCGGCCCCCTGGACAAAGACTGACGCTCAGGTG<br>CGAAAGCGTGGGGAGCAAACAGG

779	*g_Pseudomonas*	0.32	TACGAAGGGTGCAAGCGTTAATCGGAATTACTGGGCGTAAAGCGCGCGTAGGTGGTTCAGCAAGTTGGATGTGAAATCCCCGGGCTCAACCTGGGAACTGCATCCAAAACTACTGAGCTAGAGTACGGTAGAGGGTGGTGGAATTTCCTGTGTAGCGGTGAAATGCGTAGATATAGGAAGGAACACCAGTGGCGAAGGCGACCACCTGGACTGATACTGACACTGAGGTGCGAAAGCGTGGGGAGCAAACAGG
163	*g_Enterococcus*	0.32	TACGTAGGTGGCAAGCGTTGTCCGGATTTATTGGGCGTAAAGCGAGCGCAGGCGGTTTCTTAAGTCTGATGTGAAAGCCCCCGGCTCAACCGGGGAGGGTCATTGGAAACTGGGAGACTTGAGTGCAGAAGAGGAGAGTGGAATTCCATGTGTAGCGGTGAAATGCGTAGATATATGGAGGAACACCAGTGGCGAAGGCGGCTCTCTGGTCTGTAACTGACGCTGAGGCTCGAAAGCGTGGGGAGCAAACAGG
75	*g_Bacteroides*	0.29	TACGGAGGATCCGAGCGTTATCCGGATTTATTGGGTTTAAAGGGAGCGTAGGTGGACTGGTAAGTCAGTTGTGAAAGTTTGCGGCTCAACCGTAAAATTGCAGTTGATACTGTCAGTCTTGAGTACAGTAGAGGTGGGCGGAATTCGTGGTGTAGCGGTGAAATGCTTAGATATCACGAAGAACTCCGATTGCGAAGGCAGCTCACTGGACTGCAACTGACACTGATGCTCGAAAGTGTGGGTATCAAACAGG
456	*g_Veillonella*	0.29	GTGTGCCAGCCGCGGTAATACGTAGGTGGCAAGCGTTGTCCGGAATTATTGGGCGTAAAGCGCGCGCAGGCGGATCGGTCAGTCTGTCTTAAAAGTTCGGGGCTTAACCCCGTGATGGGATGGAAACTGCCAATCTAGAGTATCGGAGAGGAAAGTGGAATTCCTAGTGTAGCGGTGAAATGCGTAGATATTAGGAAGAACACCAGTGGCGAAGGCGACTTTCTGGACGAAAACTGACGCTGAGGCGCGAAAGCCAGGGGAGCGAACGGG
205	*g_Staphylococcus*	0.28	TACGTAGGTGGCAAGCGTTATCCGGAATTATTGGGCGTAAAGCGCGCGTAGGCGGTTTTTTAAGTCTGATGTGAAAGCCCACGGCTCAACCGTGGAGGGTCATTGGAAACTGTAAAACTTGAGTGCAGAAGAGGAAAGTGGAATTCCATGTGTAGCGGTGAAATGCGCAGAGATATGGAGGAACACCAGTGGCGAAGGCGACTTTCTGGTCTGTAACTGACGCTGATGTGCGAAAGCGTGGGGATCAAACAGG
538	*g_Propionimicrobium*	0.27	TACGTAGGGTGCGAGCGTTGTCCGGAATTATTGGGCGTAAAGAGCTTGTAGGCGGTTTGTCGCGTCGAAAGTGTAAACTCAGTGCTTAACGCTGAGCCTGCTTTCGATACGGGCTGACTAGAGGAAGGTAGGGGAGAATGGAATTCCCGGTGGAGCGGTGGAATGCGCAGATATCGGGAGGAACACCAGTGGCGAAGGCGGTTCTCTGGACCTTTCCTGACGCTGAGAAGCGAAAGCGTGGGTAGCAAACAGG
412	*g_Fastidiosipila*	0.27	TACGTAGGTGGCGAGCGTTATCCGGAATTACTGGGTGTAAAGGGCGTGTAGGCGGCACTGTAAGTCAGATGTGAAATCTCCCGGCTCAACCGGGAGCGTGCATCTGATACTGCAGTACTTGAGTGATAGAGGGGAAAGCGGAATTCCTAGTGTAGCGGTAAAATGCGTAGATATTAGGAGGAACACCAGTGGCGAAGGCGGCTTTCTGGCTATTAACTGACGCTGAGGCGCGAAAGCGTGGGGAGCAAACAGG
382	*g_Anaerococcus*	0.27	TACGTAAGGTCCGAGCGTTGTCCGGAATCATTGGGCGTAAAGGGTACGTAGGCGGGTAAGCAAGTTAGAAGTGAAATCCTATAGCTCAACTATAGTAAGCTTTTAAAACTGCTCATCTTGAGGTATGGAAGGGAAAGTGGAATTCCTAGTGTAGCGGTGAAATGCGCAGATATTAGGAGGAATACCGGTGGCGAAGGCGACTTTCTGGCCATAACCTGACGCTGAGGTACGAAAGCGTGGGTAGCAAACAGG
292	*g_Streptococcus*	0.26	TACGTAGGTCCCGAGCGTTGTCCGGATTTATTGGGCGTAAAGCGAGCGCAGGCGGTTAGATAAGTCTGAAGTGAAAGGCAGTGGCTCAACCATTGTAGGCTTTGGAAACTGTTTAACTTGAGTGCAGAAGGGGAGAGTGGAATTCCATGTGTAGCGGTGAAATGCGTAGATATATGGAGGAACACCGGTGGCGAAAGCGGCTCTCTGGTCTGTAACTGACGCTGAGGCTCGAAAGCGTGGGGAGCGAACAGG
